# Indacaterol/glycopyrronium versus salmeterol/fluticasone in the prevention of clinically important deterioration in COPD: results from the FLAME study

**DOI:** 10.1186/s12931-018-0830-z

**Published:** 2018-06-20

**Authors:** Antonio R. Anzueto, Konstantinos Kostikas, Karen Mezzi, Steven Shen, Michael Larbig, Francesco Patalano, Robert Fogel, Donald Banerji, Jadwiga A. Wedzicha

**Affiliations:** 1 University of Texas Health Science Center and South Texas Veterans Healthcare System, University of Texas, San Antonio, TX USA; 2 Novartis Pharma AG, Basel, Switzerland; 3 Novartis Pharmaceuticals Corporation, East Hanover, NJ USA; 4 Respiratory Clinical Science, National Heart and Lung Institute, Imperial College London, London, UK

**Keywords:** Chronic obstructive pulmonary disease, COPD, Clinically important deterioration, CID, Indacaterol/glycopyrronium, Salmeterol/fluticasone, LABA/LAMA, FLAME

## Abstract

**Background:**

Chronic obstructive pulmonary disease (COPD) is a progressive disease and a composite endpoint could be an indicator of treatment effect on disease worsening. This post-hoc analysis assessed whether indacaterol/glycopyrronium (IND/GLY) 110/50 μg once daily reduced the risk of clinically important deterioration (CID) versus salmeterol/fluticasone (SFC) 50/500 μg twice daily in moderate-to-very severe COPD patients from the FLAME study.

**Methods:**

CID was defined as ≥100 mL decrease in forced expiratory volume in 1 s (FEV_1_) or ≥ 4-unit increase in St. George’s Respiratory Questionnaire (SGRQ) total score or a moderate-to-severe COPD exacerbation. Changes from baseline in the rate of moderate and severe exacerbations, time to first moderate-to-severe exacerbation, and change from baseline in the SGRQ score, measured after Week 12 up to Week 52, were assessed by presence of early CID (CID+) or absence of CID (CID−) at Week 12.

**Results:**

IND/GLY significantly delayed the time to CID (hazard ratio [HR] (95% confidence interval [CI]), 0.72 [0.67–0.78]; *P* < 0.0001), and reduced the incidences of CID versus SFC. Additionally, IND/GLY delayed the time to CID in all patient subgroups. After 12 weeks until 52 weeks, CID+ patients had a significantly higher rate of moderate-to-severe exacerbations versus CID− patients (*P* < 0.0001); moreover, CID+ patients experienced moderate-to-severe exacerbations significantly earlier versus CID− patients (*P* < 0.0001). CID+ patients had a comparable change in the SGRQ total score versus CID− patients.

**Conclusions:**

IND/GLY reduced the risk of CID versus SFC. CID had a significant impact on long-term exacerbation outcomes in patients with moderate-to-very severe COPD and a history of ≥1 exacerbations in the previous year.

**Trial registration:**

Clinicaltrials.gov NCT01782326.

**Electronic supplementary material:**

The online version of this article (10.1186/s12931-018-0830-z) contains supplementary material, which is available to authorized users.

## Background

Chronic obstructive pulmonary disease (COPD) is a progressive disease characterized by poorly reversible airflow obstruction, gradual worsening of symptoms, deteriorating health status, and exacerbations [1]. Together, these factors are important indicators of COPD prognosis. The World Health Organization (WHO) has estimated COPD to be the fourth most common single cause of death worldwide and the treatment and management costs to present a significant burden to public health [2]. The primary goals of COPD management are to improve symptoms, exercise tolerance and health status, prevent exacerbations and disease progression and reduce mortality [1].

Randomized clinical trials of medications for COPD generally assess the efficacy of these treatments by evaluating their ability to improve COPD based on the minimum clinically important difference (MCID) between treatments [3, 4]; however, considering the progressive nature of COPD, it is also essential to evaluate these treatments for their effect on prevention of disease worsening. Composite endpoints for evaluating the effect of treatment on COPD outcomes in terms of lung function, COPD symptoms and exacerbations, health status, and quality of life may be a more appropriate way to produce a comprehensive view of the disease [5]. Clinically important deterioration (CID) is a composite endpoint that incorporates criteria related to lung function, health status or symptoms, and exacerbations, and thus can be used as an indicator of treatment effect on COPD worsening [5, 6]. CID was first used as a composite endpoint in patients with COPD in a pooled analysis of two 24-week trials that evaluated the efficacy of a combination of long-acting β_2_-agonist (LABA, vilanterol) and long-acting muscarinic-antagonist (LAMA, umeclidinium) bronchodilators [6]. This composite endpoint is consistent with the current Global Initiative for Chronic Obstructive Lung Disease (GOLD) strategy, which recommends that COPD outcomes in terms of lung function, symptoms, and health status should be considered when assessing disease progression and severity [7].

Dual bronchodilation with the fixed-dose LABA/LAMA combination of indacaterol/glycopyrronium (IND/GLY) has proven to be an effective treatment option for patients with COPD demonstrating significant improvements in lung function [8], dyspnea [9], quality of life [3], and exacerbation rates versus both the LAMA tiotropium [3], and the LABA/ICS combination, salmeterol/fluticasone (SFC), in COPD patients [8, 10, 11]. Furthermore, post-hoc analysis of pooled data from the LANTERN and ILLUMINATE studies showed that IND/GLY significantly reduced the risk of CID versus SFC in patients with moderate-to-severe COPD [5].

In this post-hoc analysis of the FLAME study, we assessed whether IND/GLY (110/50 μg once daily [o.d.]) delayed the time to CID versus SFC (50/500 μg twice daily [b.i.d.]) in patients with moderate-to-very severe COPD and a history of ≥1 exacerbations in the previous year. We also explored the predictive impact of an early CID (within the 1^st^ 12 weeks) on subsequent study outcomes.

## Methods

### Study design

**Fig. 1:**
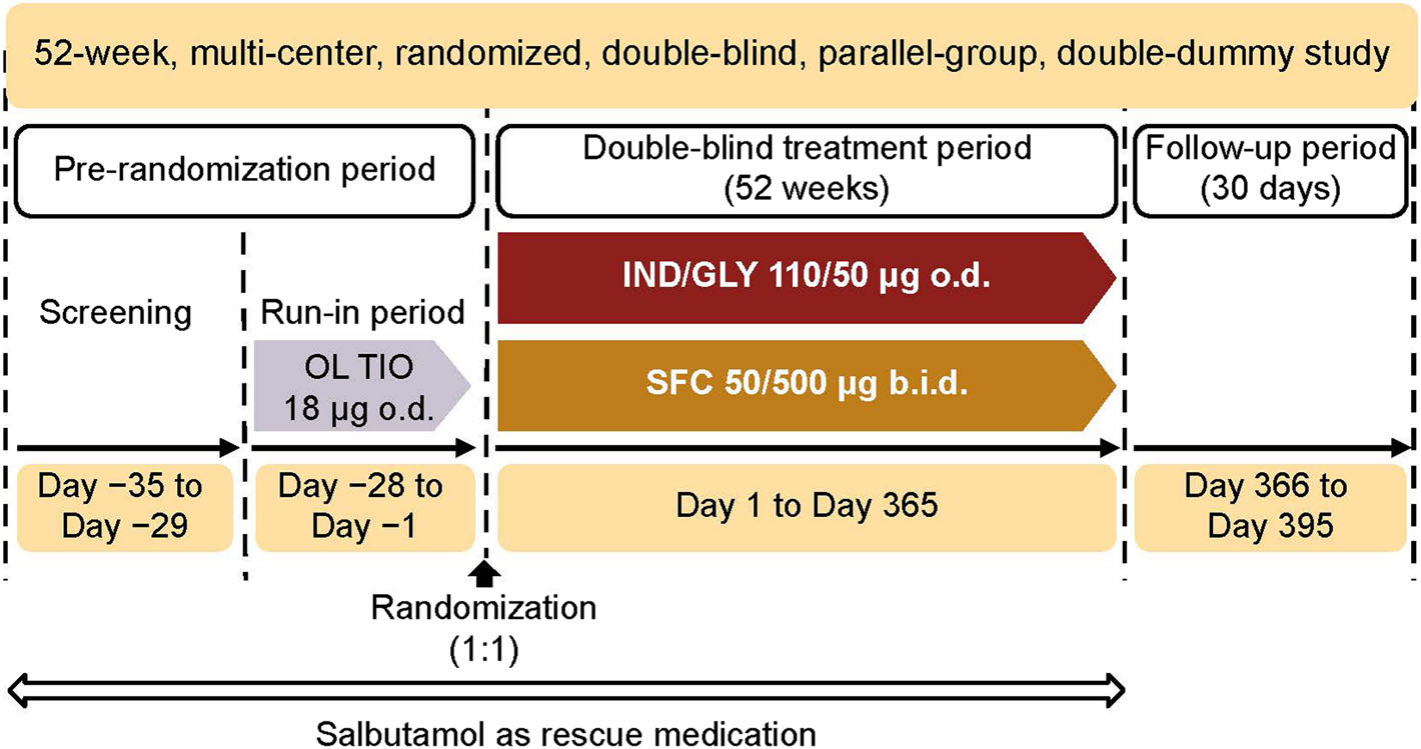
Study design. b.i.d., twice daily; IND/GLY, indacaterol/glycopyrronium; o.d., once daily; OL, open-label; SFC, salmeterol/fluticasone; TIO, tiotropium

Details of the FLAME study design have been reported previously [10]. Briefly, FLAME (NCT01782326) was a Phase III, 52-week, multicenter, randomized, double-blind, double-dummy, parallel-group study. Following the 1-week screening and 4-week run-in periods, patients were randomized to receive either IND/GLY 110/50 μg o.d. or SFC 50/500 μg b.i.d. for 52 weeks, with an additional 30-day follow-up period (Fig. 1). The study was conducted according to the ethical principles of the Declaration of Helsinki, and all patients provided written informed consent.

### Patients

Patients aged ≥40 years with a post-bronchodilator forced expiratory volume in 1 s (FEV_1_) ≥25 and < 60% predicted, a documented history of ≥1 COPD exacerbation (for which they received treatment with systemic corticosteroids and/or antibiotics) in the previous 12 months, and modified Medical Research Council (mMRC) dyspnea scale grade ≥ 2 were included in the FLAME study. Full inclusion and exclusion criteria have been described previously [10].

### Definitions and assessment of CID

The risk of CID was assessed using definitions of CID based on the MCID for the various endpoints, as described previously by Singh et al. [6]. CID was defined as any of the following: a ≥ 100 mL decrease from baseline in pre-dose FEV_1_, a ≥ 4-unit increase in St. George’s Respiratory Questionnaire (SGRQ) total score from baseline, or a moderate-to-severe COPD exacerbation occurring after the first dose of study medication. The time to CID was also assessed and defined as the first time points at which CID occurred.

In order to assess the predictive value of early clinical deterioration, the presence (CID+) or absence of CID (CID–) at Week 12 was also evaluated. The rates of moderate and severe exacerbations, time to first moderate-to-severe exacerbation, and change from baseline in the SGRQ score measured after Week 12 up to Week 52 by presence of early CID (CID+) or absence of CID (CID−) at Week 12 were assessed.

### Statistical analyses

All analyses were performed on the full analysis set, defined as patients who were randomized and received at least one dose of the study treatment. Descriptive statistics (n and percentage) were used to summarize the events of CID. Statistical comparisons of IND/GLY versus SFC were conducted for CID per definition. Kaplan–Meier curves were generated for the time-to-event data, and hazard ratios (HRs) with 95% confidence interval (CI) of the time to event were estimated and compared by using the Cox Proportional Hazard model. Covariates included in the model were treatment group, gender, age group, baseline COPD severity, ex-smoker (yes/no), and eosinophil count at baseline (≥300 or < 300 cells/μL). In addition, log rank tests were used to compare the Kaplan–Meier curves for treatment comparisons, with *P* values presented alongside Kaplan–Meier curves. For time to CID analysis, patients without an event who remained on treatment were censored at the study end date; those who had discontinued were censored at their last study contact date. Subgroup analyses were performed for each of the endpoints to explore the consistency of the overall treatment effect on the time to CID; the subgroups used were: gender, age (≥65 versus < 65 years), baseline COPD severity (moderate, severe, or very severe), smoking status (ex-smokers versus current smokers), and baseline blood eosinophil count (≥300 versus < 300 cells/μL). The concordance between the different criteria for CID was evaluated using kappa statistics.

## Results

### Patients

Of the 3362 patients (IND/GLY, 1680; SFC, 1682) randomized in the FLAME study, 82.1% completed 52 weeks of treatment. Patient demographics and other baseline characteristics were generally similar between the treatment groups (Table 1). A total of 19.3% of patients had a history of two or more moderate or severe exacerbations during the previous year. There were no differences between treatment groups in baseline lung function, bronchodilator reversibility, or the proportion of GOLD D patients (GOLD 2015 criteria).

**Table 1:** Baseline demographics and clinical characteristics (randomized set)

Characteristics	Indacaterol/glycopyrronium 110/50 μg o.d.	Salmeterol/fluticasone 50/500 μg b.i.d.
	(*n* = 1680)	(*n* = 1682)
Age, years	64.6 ± 7.89	64.5 ± 7.70
Men, *n* (%)	1299 (77.3)	1258 (74.8)
COPD severity^a^, *n* (%)		
Moderate, GOLD 2	560 (33.3)	563 (33.5)
Severe, GOLD 3	973 (57.9)	981 (58.3)
Very severe, GOLD 4	133 (7.9)	124 (7.4)
High risk and more symptoms (Group D)	1265 (75.3)	1249 (74.3)
Current smokers, *n* (%)	664 (39.5)	669 (39.8)
Number of COPD exacerbations in the previous year, *n* (%)		
1	1355 (80.7)	1355 (80.6)
≥ 2	324 (19.3)	325 (19.3)
SGRQ-C total score^b^	47.3 (15.8)	47.2 (15.9)
Post-bronchodilator FEV_1_, L	1.2 ± 0.34	1.2 ± 0.35
Post-bronchodilator FEV_1_, % predicted	44.0 ± 9.48	44.1 ± 9.43
Post-bronchodilator FEV_1_/FVC, %	41.7 ± 9.82	41.5 ± 9.89

Data presented as mean ± SD, unless otherwise specified; ^a^COPD severity is based on the GOLD 2015 criteria [27]; GOLD 2011 [28]; ^b^On a scale of 0–100, with higher scores indicating worse health status; the MCID is a change of 4 units; On a scale of 0–4, with higher scores indicating more severe dyspnea; On a scale of 0–40, with higher scores indicating worse health status

b.i.d., twice daily; CAT, COPD assessment test; COPD, chronic obstructive pulmonary disease; FEV_1,_ forced expiratory volume in 1 s; FVC, forced vital capacity; GOLD, Global Initiative for Chronic Obstructive Lung Disease; ICS, inhaled corticosteroid; MCID, minimum clinically important difference; mMRC, modified Medical Research Council; o.d., once daily; SD, standard deviation; SGRQ-C, St. George’s Respiratory Questionnaire for COPD

### Components of the composite CID endpoint

**Fig. 2:**
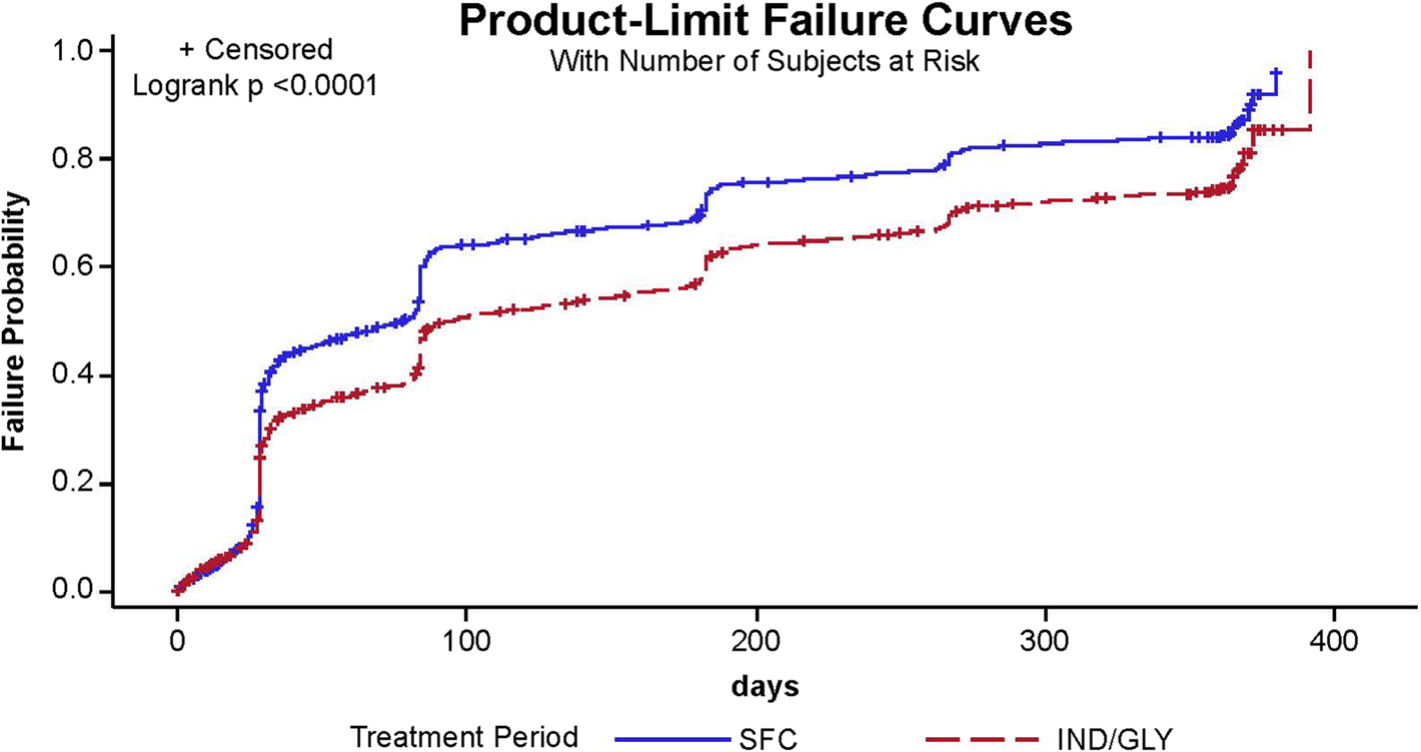
Kaplan–Meier curves and Cox proportional hazard-model for time-to CID during 52 weeks of treatment. b.i.d., twice daily; CID, clinically important deterioration; IND/GLY, indacaterol/glycopyrronium; o.d., once daily; SFC, salmeterol/fluticasone

IND/GLY significantly delayed the time to CID versus SFC, reducing the risk of CID by 28% versus SFC (HR, 0.72; 95% CI, 0.67 to 0.78; *P* < 0.0001; Fig. 2). Data on the time course of the individual CID components, the number of patients with an event and time to first event for each individual CID component (moderate or severe exacerbation, SGRQ deterioration or trough FEV_1_ deterioration) are provided in the Additional file 1: Figures S1–S3 and Tables S1-S3). Differences in all components were observed in favor of IND/GLY. In an evaluation of the concordance of CID events using kappa statistics, the results suggest no or minimal concordance between the different criteria, as all kappa values were < 0.20 for each pair of CID events (Additional file 1: Table S4).

### Subgroup analysis

**Fig. 3:**
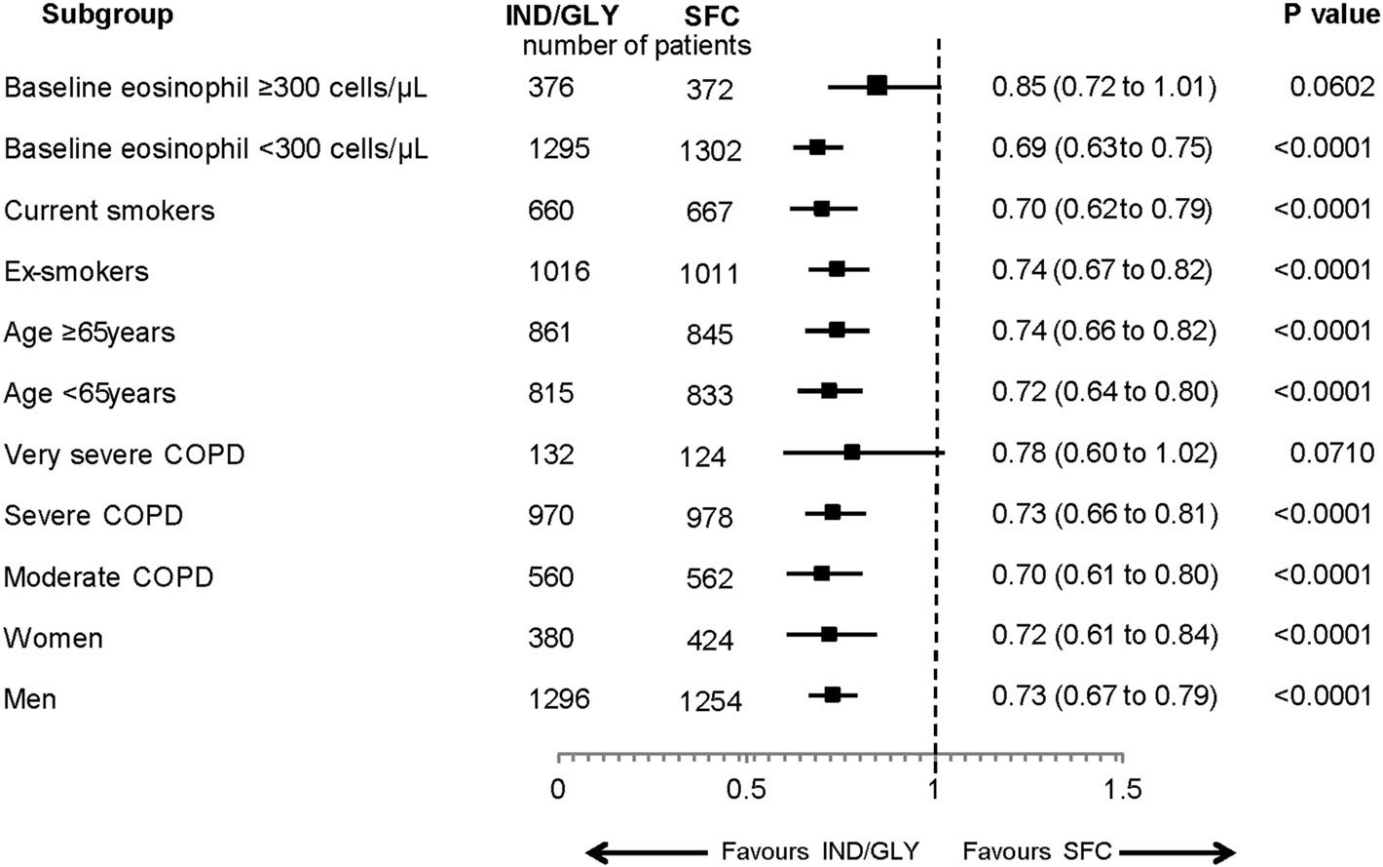
Hazard ratios and respective 95% CI for time-to CID by subgroup during 52 weeks of treatment. b.i.d., twice daily; CI, confidence interval; CID, clinically important deterioration; COPD, chronic obstructive pulmonary disease; HR, hazard ratio; IND/GLY, indacaterol/glycopyrronium 110/50 μg o.d.; o.d., once daily; SFC, salmeterol/fluticasone 50/500 μg b.i.d

Among the subgroups evaluated, IND/GLY significantly reduced the risk of CID versus SFC in all subgroups; in the groups of patients with baseline eosinophil count ≥300 cells/μL and those with very severe COPD, numerical reductions in the risk were observed, that did not reach statistical significance (Fig. 3).

### COPD exacerbations and health status

Of the 3354 patients, 1853 (55.2%) experienced CID, while 1501 (44.8%) did not by 12 weeks. Between 12 and 52 weeks, CID+ patients had a significantly higher rate of moderate-to-severe exacerbations versus CID− patients (*P* < 0.0001, Table 2). CID+ patients had a significantly higher risk for the time to first moderate-to-severe exacerbation than CID− patients (*P* < 0.0001; Table 2). Change in the SGRQ total score in CID+ patients was comparable with that in CID− patients (Table 2).

**Table 2:** Exacerbations and quality-of-life outcomes after 12 weeks until 52 weeks according to occurrence of CID

Outcome	CID+ versus CID−
Moderate-to-severe exacerbations	RR: 1.8 (1.60 to 2.02); *P* < 0.0001
(N_1_ = 1615; N_2_ = 1334)	
Time to first moderate-to-severe exacerbations (N_1_ = 1615; N_2_ = 1334)	HR: 2.24 (2.02 to 2.50); *P* < 0.0001
LSM change in SGRQ total score	−0.59 (−1.43 to 0.25); *P* = 0.1697
(N_1_ = 1600; N_2_ = 1336)	

*CID* clinically important deterioration, *CID*+ presence of CID at Week 12, *CID*− absence of CID at Week 12, *HR* hazard ratio, *LSM* least squares mean, *N*_1_ number of patients in the CID+ group, *N*_2_ number of patients in the CID− group, *RR* rate ratio, *SGRQ* St. George’s Respiratory Questionnaire

## Discussion

This analysis demonstrated that in patients with moderate-to-very severe COPD and a history of ≥1 exacerbations in the previous year, the dual bronchodilator IND/GLY significantly reduced the risk of a CID compared with SFC. These results were consistent in the subgroup analyses based on baseline patient demographics and clinical characteristics. This is the first evaluation of the concept of CID between LABA/LAMA and LABA/ICS in a population of exacerbating COPD patients.

COPD is a progressive disease, and prevention of disease worsening is one of the major goals in COPD management [1]. Moreover, evaluation of disease progression can assist clinicians in choosing the most appropriate treatment for patients [5]. Analysis of the effectiveness of a treatment using a composite endpoint is now widely accepted in clinical trials of complex diseases, including cardiovascular diseases and neoplasia, assuming that the individual components of the composite endpoint are of clinical importance [12]. The effective use of composite endpoints may increase the efficiency of clinical trials by reducing sample sizes, costs, and time. These endpoints may also help investigators identify outcomes that refer to disease progression and facilitate the assessment of patient-reported outcomes that provide information on multiple aspects of the patients’ perceptions of their health status [13]. The use of composite tools for the assessment of COPD progression has been reported previously. The Body-mass index, airflow Obstruction, Dyspnea, and Exercise capacity (BODE) was one of the first composite indexes to evaluate patients’ physiological, physical, and clinical aspects; several other composite indexes have been used for the evaluation of COPD severity and outcomes [14–18]. In the present analysis, the use of CID as a composite endpoint for COPD was based on lung function, health status, and exacerbations, all of which contribute to the long-term prognosis of the disease.

In this study, lung function and time to first moderate or severe exacerbation were the strongest drivers of CID. The time to first significant decline in FEV_1_ and time to first moderate or severe exacerbation events were significantly longer in IND/GLY-treated versus SFC-treated patients. This finding is in line with previously published results in which IND/GLY significantly reduced the risk of CID versus tiotropium and SFC in patients with moderate-to-severe COPD [5]. Furthermore, CID was also significantly delayed in the IND/GLY group versus the SFC treatment group (*P* < 0.05) based on the SGRQ definition. The concordance between different CID events was very low (or even absent), suggesting that these individual events contribute independently to the deterioration of patients at certain time points. An important limitation of this analysis (that is likely universal to all CID evaluations in clinical trials) is that the FEV_1_ and SGRQ were only collected at certain time points, in contrast to the continuous collection of exacerbation events during the 1-year follow-up. These findings also suggest that dual bronchodilation with IND/GLY provides statistically significant improvements in lung function and health status compared with SFC, and importantly, provide greater protection against future deterioration.

Several demographic characteristics, including age, gender, airflow limitation, smoking history, and baseline eosinophil count have been evaluated as factors that may have an impact on the treatment responsiveness of patients with COPD [19–24]. Findings from the subgroup analysis, based on demographics and baseline characteristics, are in line with the overall results of the study. IND/GLY significantly reduced the risk of CID in all subgroups except patients with baseline eosinophil ≥300 cells/μL and in those with very severe COPD, although the number of patients with very severe COPD is small. The outcomes of our analysis support a previous study in which IND/GLY significantly delayed the first occurrence of CID versus tiotropium and SFC in patients with moderate-to-severe COPD [5]. Outcomes of the present analysis, together with other published studies, show that IND/GLY can prevent CID in patients with COPD irrespective of disease severity [5]. It further justified the selection of individual parameters by showing stability of the components and precision of the process. These analyses add to the existing repertoire of CID definitions applied to COPD trials, which may aid in determining the most applicable definition(s) of CID endpoint for COPD to be used in routine clinical settings.

COPD is characterized by progressive decline in lung function, and this decline is accelerated by exacerbations [25]. This study demonstrated that CID+ patients (i.e., those who experienced CID by Week 12) had significantly higher rate and risk for time to first moderate or severe exacerbation compared with CID− patients in the following 40 weeks. This finding was not unexpected given that CID represents a period of disease worsening [6], and the CID+ patients were in a more severe stage of the disease at Week 12 compared with the CID− patients. These data are in line with the previously reported post-hoc analysis from the TORCH and ECLIPSE studies, where lung function, health status, and exacerbation risk were worse in patients who experienced an early CID [26]. However, in our study despite a numerical difference in favor of the CID- patients, the change in SGRQ was comparable between the CID+ and CID− populations. This may be attributed to the fact that the follow-up time for SGRQ was only 40 weeks and this may have contributed to the smaller between-treatment differences. Another possible reason can be that active treatments were used in both arms of this study and this may have had an impact on the observed differences.

Some limitations of these results must be acknowledged. These are secondary, post-hoc analyses of the FLAME study and some subgroup analyses involved small sample sizes or fewer events and should, therefore, be interpreted with caution. Nonetheless, the observed trends consistently favored IND/GLY across multiple analyses, supporting the primary results of the FLAME study and providing further reassurance that the beneficial effects of IND/GLY compared with SFC are not limited to specific subpopulations. Furthermore, the creation of the CID composite endpoint required evaluation of each included variable, which was performed with a high degree of rigor and hence the overall findings are robust.

## Conclusion

This analysis demonstrated that IND/GLY reduced the risk of CID of COPD compared with SFC, thus providing sustained efficacy in symptomatic patients with moderate-to-very severe COPD and a history of ≥1 exacerbations in the previous year. These results further support the use of IND/GLY as a first-line steroid-free treatment option for patients with moderate-to-very severe COPD with a history of ≥1 exacerbations in the previous year. Future clinical trials that prospectively validate CID are also needed to determine its clinical utility.

## Additional file

Additional file 1: **Fig S1.** Kaplan–Meier curves and Cox proportional hazard model on time to first moderate-to-severe COPD exacerbation after the first dose of CID. **Fig S2**. Kaplan–Meier curves and Cox proportional hazard model on SGRQ total score from baseline of CID. **Fig S3.** Kaplan–Meier curves and Cox proportional hazard model on pre-dose FEV_1_ of CID. **Table S1.** Summary statistics Kaplan–Meier and Cox proportional hazard model analysis on time to first moderate-to-severe COPD exacerbation from baseline. **Table S2.** Summary statistics, Kaplan–Meier and Cox proportional hazard model analysis on first SGRQ total score deterioration from baseline. **Table S3.** Summary statistics, Kaplan–Meier and Cox proportional hazard model analysis on first trough FEV_1_ deterioration from baseline. **Table S4.** Kappa statistics between events in first CID. **Table S5.** List of institutional review boards or ethics committees. (DOCX 293 kb)

## Abbreviations

b.i.d.: twice daily
CI: Confidence interval
CID: Clinically important deterioration
COPD: Chronic obstructive pulmonary disease
FEV_1_: Forced expiratory volume in 1 s
GOLD: Global Initiative for Chronic Obstructive Lung Disease
HR: Hazard ratio
ICS: Inhaled corticosteroid
IND/GLY: Indacaterol/glycopyrronium
LABA: Long-acting β_2_-agonist
LAMA: Long-acting muscarinic antagonist
MCID: Minimum clinically important difference
mMRC: Modified Medical Research Council
o.d.: Once daily
SFC: Salmeterol/fluticasone
SGRQ: St. George’s Respiratory Questionnaire
WHO: World Health Organization
