# Supplementary material for: Indacaterol/glycopyrronium versus salmeterol/fluticasone in the prevention of clinically important deterioration in COPD: results from the FLAME study
Source: Respir Res. 2018 Jun 20;19:121. doi: 10.1186/s12931-018-0830-z (PMC6011394; doi:10.1186/s12931-018-0830-z)
Supplement: Supplementary file 1 — Fig S1. Kaplan–Meier curves and Cox proportional hazard model on time to first moderate-to-severe COPD exacerbation after the first dose of CID. Fig S2. Kaplan–Meier curves and Cox proportional hazard model on SGRQ total score from baseline of CID. Fig S3. Kaplan–Meier curves and Cox proportional hazard model on pre-dose FEV1 of CID. Table S1. Summary statistics Kaplan–Meier and Cox proportional hazard model analysis on time to first moderate-to-severe COPD exacerbation from baseline. Table S2. Summary statistics, Kaplan–Meier and Cox proportional hazard model analysis on first SGRQ total score deterioration from baseline. Table S3. Summary statistics, Kaplan–Meier and Cox proportional hazard model analysis on first trough FEV1 deterioration from baseline. Table S4. Kappa statistics between events in first CID. Table S5. List of institutional review boards or ethics committees. (DOCX 293 kb) [file 12931_2018_830_MOESM1_ESM.docx]

**SUPPLEMENTARY APPENDIX**

Indacaterol/glycopyrronium versus salmeterol/fluticasone in the prevention of clinically important deterioration in COPD: results from the FLAME study

Antonio R. Anzueto, Konstantinos Kostikas, Karen Mezzi, Steven Shen, Michael Larbig, Francesco Patalano, Robert Fogel, Donald Banerji and Jadwiga A Wedzicha

**SUPPLEMENTARY FIGURES**

**Supplementary Figure. 1** Kaplan–Meier curves and Cox proportional hazard model on time to first moderate-to-severe COPD exacerbation after the first dose of CID


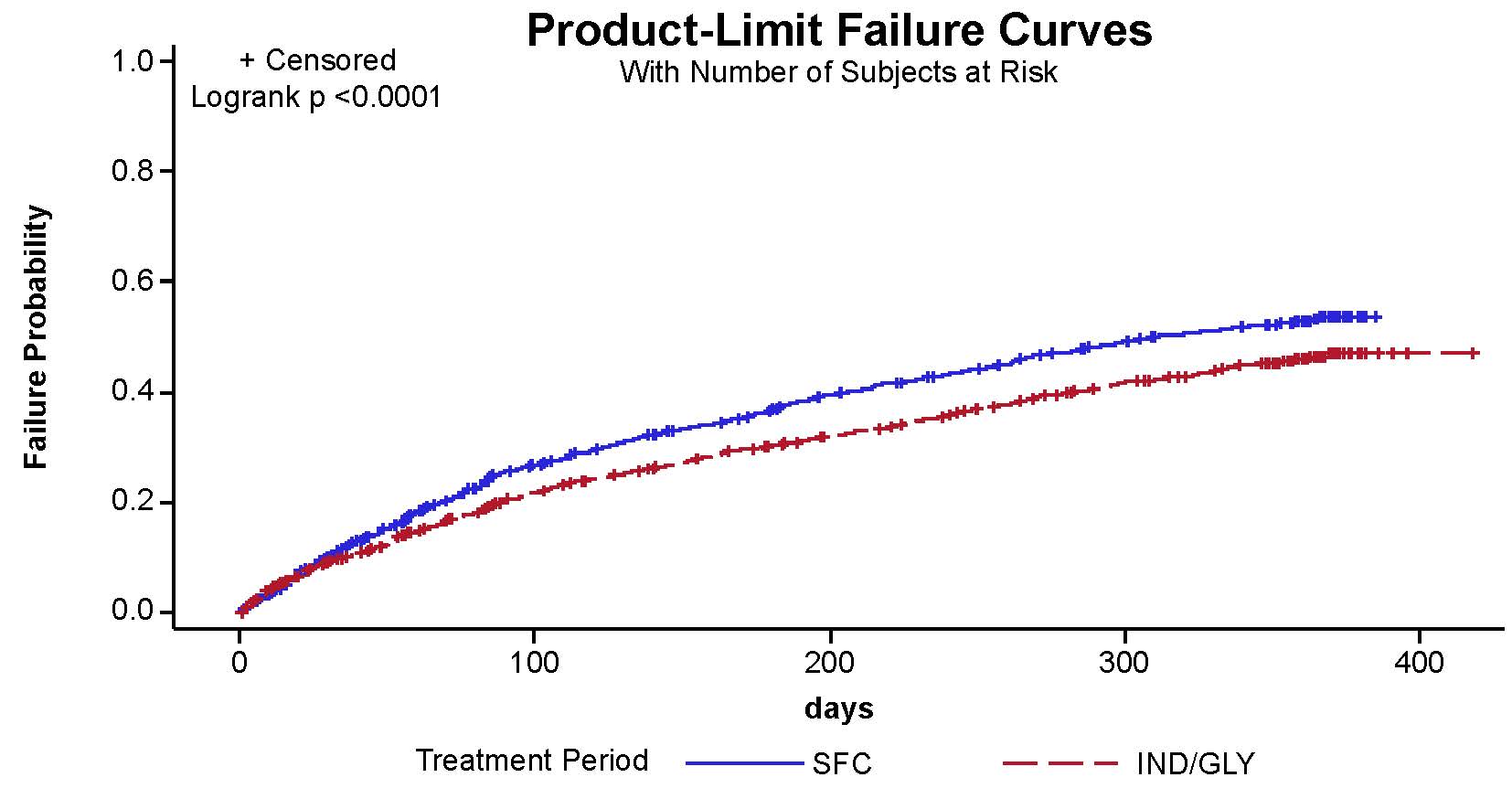


CID, clinically important deterioration; IND/GLY, indacaterol/glycopyrronium; SFC, salmeterol/fluticasone

**Supplementary Figure. 2** Kaplan–Meier curves and Cox proportional hazard model on SGRQ total score from baseline of CID


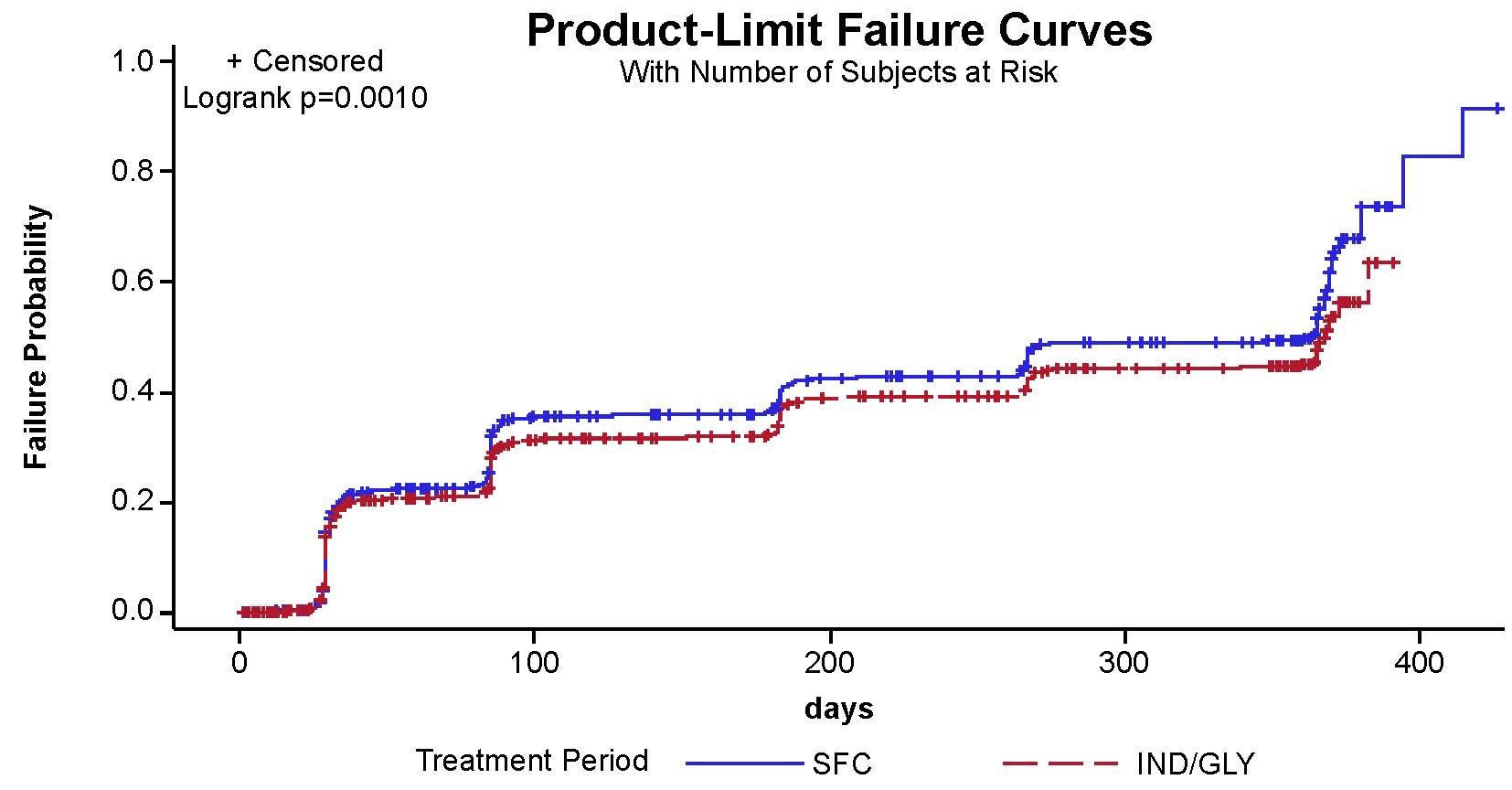


CID, clinically important deterioration; IND/GLY, indacaterol/glycopyrronium; SFC, salmeterol/fluticasone; SGRQ, St. George’s Respiratory Questionnaire

**Supplementary Figure. 3** Kaplan–Meier curves and Cox proportional hazard model on pre-dose FEV_1_ of CID


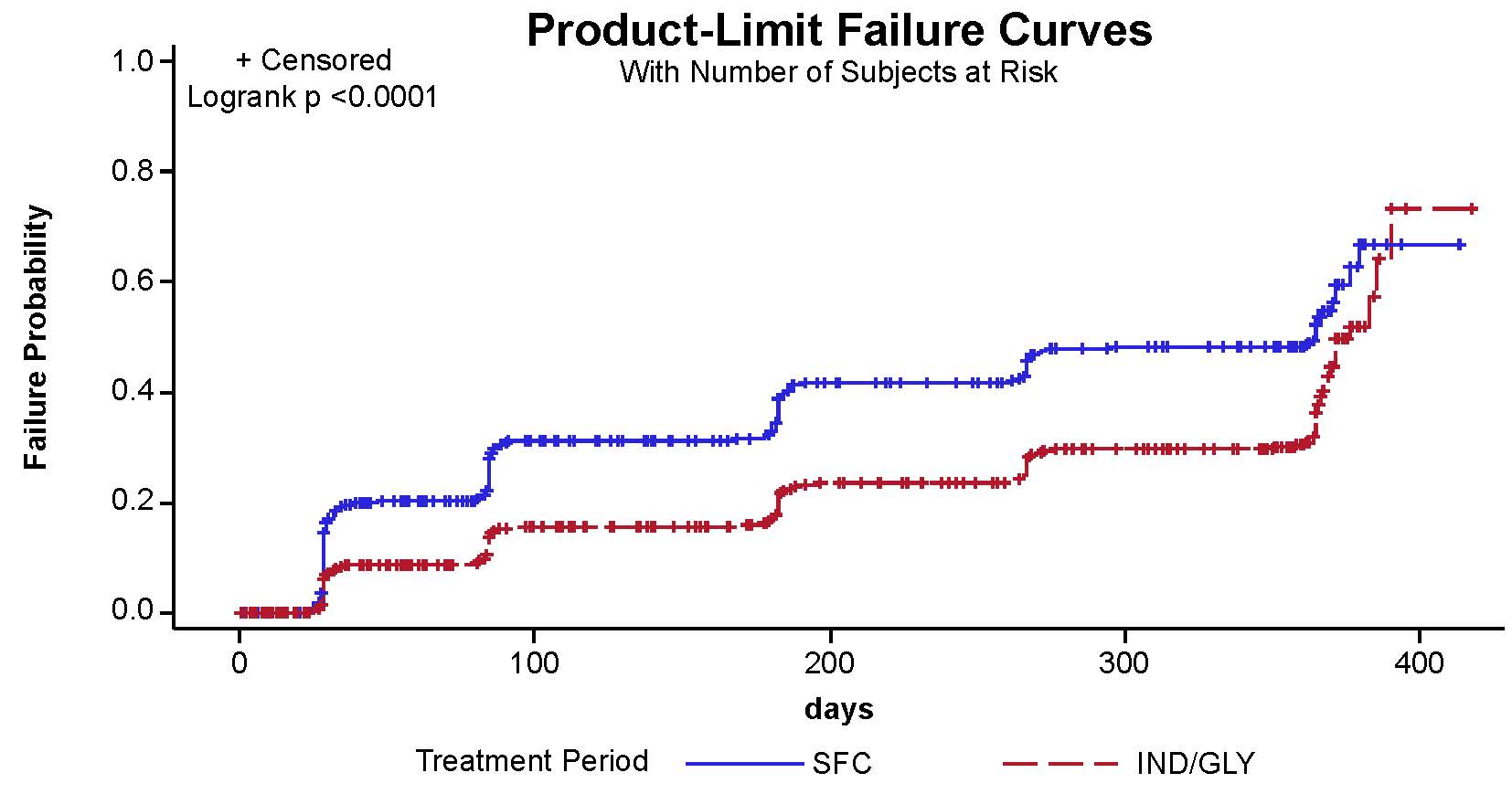


CID, clinically important deterioration; FEV_1,_ forced expiratory volume in 1 second; IND/GLY, indacaterol/glycopyrronium; SFC, salmeterol/fluticasone

**Table 1.** Summary statistics Kaplan–Meier and Cox proportional hazard model analysis on time to first moderate-to-severe COPD exacerbation from baseline

| **Time to the CID (days)** | **IND/GLY**  **N = 1676** | **SFC**  **N = 1678** |
| --- | --- | --- |
| Number of subjects with events, n (%) | 738 (44.0) | 843 (50.2) |
| 25% quartile time to first event (95% CI) (days) | 127.00 (107.00, 148.00) | 87.00 ( 82.00, 103.00) |
| Median time to first event (95% CI)(days) | NE (NE, NE) | 308.00 (283.00, 352.00) |
| CID, clinically important deterioration; IND/GLY, indacaterol/glycopyrronium; NE, not evaluated; SFC, salmeterol/fluticasone | | |

**Table 2.** Summary statistics, Kaplan–Meier and Cox proportional hazard model analysis on first SGRQ total score deterioration from baseline

| **Time to the CID (days)** | **IND/GLY**  **N = 1676** | **SFC**  **N = 1678** |
| --- | --- | --- |
| Number of subjects with events, n (%) | 762 (45.5) | 865 (51.5) |
| 25% quartile time to first event (95% CI) (days) | 85.00 (NE, NE) | 84.00 ( 80.00, 85.00) |
| Median time to first event (95% CI)(days) | 368.00 (365.00, 372.00) | 364.00 (268.00, 365.00) |
| CID, clinically important deterioration; IND/GLY, indacaterol/glycopyrronium; NE, not evaluated; SFC, salmeterol/fluticasone | | |

**Table 3.** Summary statistics, Kaplan–Meier and Cox proportional hazard model analysis on first trough FEV_1_ deterioration from baseline

| **Time to the CID (days)** | **IND/GLY**  **N = 1676** | **SFC**  **N = 1678** |
| --- | --- | --- |
| Number of subjects with events, n (%) | 560 (33.4) | 801 (47.7) |
| 25% quartile time to first event (95% CI) (days) | 266.00 (188.00, 267.00) | 85.00 (NE, NE) |
| Median time to first event (95% CI)(days) | 377.00 (372.00, 391.00) | 365.00 (273.00, 366.00) |
| CID, clinically important deterioration; IND/GLY, indacaterol/glycopyrronium; NE, not evaluated; SFC, salmeterol/fluticasone | | |

**Table 4.** Kappa statistics between events in first CID

| **CID event criterion** | **≥4 point increase in SGRQ total score from baseline** | | |
| --- | --- | --- | --- |
|  | **IND/GLY** | **SFC** | **Total** |
| ≥100 ml decrease from baseline in pre-dose FEV_1_ | 0.0994 | 0.1359 | 0.1257 |
|  | **Moderate or severe COPD exacerbation from baseline** | | |
|  | **IND/GLY** | **SFC** | **Total** |
| ≥100 ml decrease from baseline in pre-dose FEV_1_ | 0.1004 | 0.0419 | 0.0789 |
| ≥4 point increase from baseline in SGRQ total score | 0.1700 | 0.1608 | 0.1685 |
| CID is defined as the first occurrence of one of the above three components.  CID, clinically important deterioration; FEV_1_, forced expiratory volume in 1 second; IND/GLY, indacaterol/glycopyrronium 110/50 μg o.d; SFC, salmeterol/fluticasone 50/500 μg b.i.d.; SFC, salmeterol/fluticasone; SGRQ, St. George’s Respiratory Questionnaire | | | |

**Table 5.** List of institutional review boards or ethics committees.

| **#** | **EC/IRB country** | **Site Number(s)** | **EC/IRB Name** | **EC/IRB Department** | **EC/IRB City (State/Province)** |
| --- | --- | --- | --- | --- | --- |
|  | Argentina | 4003;#4008;#4011;#4014;#4017;#4019;#4023;#4027;#4029 | Comite Independiente de Etica para Ensayos en Farmacologia Clinica | NA | CABA  (Buenos Aires) |
|  | Argentina | 4001;#4002;#4004;#4007;#4013;#4018;#4021;#4025 | Comite de Etica en Investigacion Clinica | NA | CABA  (Buenos Aires) |
|  | Argentina | 4005 | Comite Independiente de etica para ensayos en farmacologia clinica del centro medico Dra. De Salvo | NA | CABA  (Buenos Aires) |
|  | Argentina | 4010 | Comite de etica en Investigaciones Biomedicas Sanatorio Otamendi y Miroli S.A | NA | CABA  (Buenos Aires) |
|  | Argentina | 4016 | Comite de Bioetica CIMEL | NA | Lanus Este  (Buenos Aires) |
|  | Argentina | 4020 | Comite de Etica en Inestigacion | NA | CABA  (Buenos Aires) |
|  | Argentina | 4022 | Comite de Etica en Investigacion Instituto de Investigaciones Clinicas | NA | Mar del Plata  (Buenos Aires) |
|  | Argentina | 4024;#4028 | Comite de Etica en Investigacion Instituto Ave Pulmo | NA | Mar del Plata  (Buenos Aires) |
|  | Argentina | 4030 | Comite de Etica en Investigacion de INAER | NA | CABA  (Buenos Aires) |
|  | Austria | 4110;#4111;#4112;#4113;#4114;#4115;#4116;#4117;#4118;#4119 | Ethikkommission des Landes Oberösterreich |  | Linz  (Oberösterreich) |
|  | Belgium | 4140;#4141;#4142;#4143;#4145;#4146;#4147;#4148;#4149;#4150;#4151;#4152;#4153;#4155;#4156;#4159;#4160;#4161 | Commissie Medische Ethiek UZ Brussel |  | Brussels |
|  | Bulgaria | 4200;#4201;#4202;#4203;#4204;#4205;#4206;#4207;#4208;#4209;#4210 | Ethics Committee for Multicenter Trials |  | Sofia |
|  | Canada | 4232;#4233;#4234;#4247;#4236;#4238;#4245;#4243;#4246;#4244 | Institutional Review Board Services |  | Aurora  (Ontario) |
|  | Canada | 4239 | Comité d’éthique de la recherché sur l’humain du Centre hospitalier universitaire de Sherbrooke |  | Fleurimont  (Quebec) |
|  | Canada | 4235 | Conjoint Health Research Ethics Board | Research Services Office | Calgary  (Alberta) |
|  | Chile | 4263 | Comité Ético Científico Hospital Dr. Gustavo Fricke. Servicio de Salud Viña del Mar - Quillota |  | Viña del Mar |
|  | Chile | 4261 | Comité de Ética Científico Servicio de Salud Metropolitano Oriente |  | Santiago, Providencia  (Región Metropolitana) |
|  | Chile | 4266 | Comité Ético Científico Servicio de Salun Metropolitano Sur |  | Santiago, San Miguel  (Región Metropolitana) |
|  | China | 4300 | Ethical Committee of the First Affiliated Hospital of Guangzhou Medical University |  | Guangzhou  (Guangdong Province) |
|  | China | 4302 | Ethical Committee of the First Affiliated Hospital of Chinese PLA General Hospital |  | Beijing  (Beijing) |
|  | China | 4301 | Ethical Committee of the General Hospital of Guangzhou Military Command of PLA |  | Guangzhou  (Guangdong Province) |
|  | China | 4303 | Ethical Committee of Beijing Friendship Hospital, Capital Medical University |  | Beijing  (Beijing) |
|  | China | 4304 | Ethical Committee of Peking Union Medical College Hospital |  | Beijing  (Beijing Province) |
|  | China | 4305 | Ethical Committee of The Third Xiangya Hospital of Central South University |  | Changsha  (Hunan Province) |
|  | China | 4306 | Ethical Committee of West China Hospital of Sichuan University |  | Chengdu  (Sichuan) |
|  | China | 4307 | Ethical Committee of Southwest Hospital, the Third Military Medical University |  | Chongqing  (Chongqing) |
|  | China | 4308 | Ethical Committee of The Second Affiliated Hospital of Third Military Medical University |  | Chongqing  (Chongqing) |
|  | China | 4309 | Ethical Committee of Hainan provincial people’s Hospital |  | Haikou  (Hainan Provience) |
|  | China | 4310 | Ethical Committee of Hangzhou First People’s Hospital |  | Hangzhou  (Zhejiang Province) |
|  | China | 4312 | Ethical Committee of The Jiangxi Provincial People's Hospital |  | Nanchang  (Jiangxi Provience) |
|  | China | 4313 | Ethical Committee of The First Affiliated Hospital of Nanchang University |  | Nanchang  (Jiangxi Provience) |
|  | China | 4315 | Ethical Committee of Shanghai Pulmonary Hospital |  | Shanghai  (Shanghai) |
|  | China | 4316 | Ethical Committee of Ruijin Hospital Affiliated to Shanghai JiaoTong University School of Medicine |  | Shanghai  (Shanghai) |
|  | China | 4317 | Ethical Committee of The General Hospital of Shenyang Military Region |  | Shenyang  (Liaoming Provience) |
|  | China | 4318 | Ethical Committee of The Second Hospital of Hebei Medical University |  | Shijiazhuang  (Hebei Province) |
|  | China | 4320 | Ethical Committee of The First Affiliated Hospital of Soochow University |  | Suzhou  (Jiangsu Provience) |
|  | China | 4321 | Ethical Committee of The General Hospital Tianjin Medical University |  | Tianjin  (Tianjin) |
|  | China | 4322 | Ethical Committee of The First Affiliated Hospital of the Fourth Military Medical University |  | Xi'an  (Shanxi Provience) |
|  | China | 4323 | Ethical Committee of Jiangsu Province Hospital |  | Nanjing  (Jiangsu Province) |
|  | Colombia | 4400 | Comité de ética en Investigación - Fundación Cardiomet Eje Cafetero | NA | Manizales  (Caldas) |
|  | Colombia | 4401 | Comité de Etica de la investigación CAIMED | NA | Bogotá  (Cundinamarca) |
|  | Colombia | 4402 | Comité Medico, Etico, Científico Asesor - Clínica Soma | NA | Medellín  (Antioquia) |
|  | Colombia | 4403 | Comité de ética en Investigación CEI- FOSCAL | NA | Floridablanca  (Santander) |
|  | Croatia | 1770;#1771;#1772 | SEP - Sredisnje eticko povjerenstvo |  | Zagreb |
|  | Czech Republic | 4420;#4421;#4422;#4423;#4424;#4426;#4427;#4432;#4425;#4436 | Etická komise IKEM a TN | Thomayerova nemocnice | Praha 4 -Krc |
|  | Czech Republic | 4430;#4435 | Eticka komise | Vitkovicka nemocnice a.s. | Ostrava - Vitkovice |
|  | Czech Republic | 4433 | Eticka komise | Nemocnice Melnik | Melnik |
|  | Czech Republic | 4434 | Eticka komise | Nemocnice Rudolfa a Stefanie Benešov, a.s. | Benešov |
|  | Czech Republic | 4437 | Etiicka komise FN a LF UP Olomouc |  | Olomouc |
|  | Czech Republic | 4428;#4429;#4431 | Etická komise IKEM a TN | Thomayerova nemocnice | Praha 4 - Krc |
|  | Denmark | 4460;#4461;#4462;#4463;#4464;#4465;#4466;#4467;#4468 | De Videnskabsetiske Komiteer | Region Hovedstaden | Hillerød |
|  | Estonia | 4500;#4501;#4502 | Tallinn Medical Research Ethics Committee | NA | Tallinn  (NA) |
|  | Finland | All sites/central Vote;#4520;#4521;#4523;#4524;#4525;#4526 | Keski-Suomen sairaanhoitopiirin eettinen toimikunta |  | Jyväskylä |
|  | France | 4540;#4541;#4542;#4543;#4544;#4545;#4546;#4548;#4551;#4552;#4554;#4555 | CPP Ile de France XI | Centre Hospitalier de Saint-Germain-Laye | Saint Germain en Laye Cedex  (NA) |
|  | Germany | All sites/central Vote | Ethikkommission Medizinische Fakultät Heidelberg | Medizinische Fakultät Heidelberg | Heidelberg  (Baden-Württemberg) |
|  | Greece | 4710;#4711;#4712;#4713;#4714;#4715;#4717;#4718;#4719;#4720;#4721 | National Ethics Committee (EED) |  | Cholargos  (Attiki) |
|  | Guatemala | 4740;#4741;#4742;#4743;#4745 | Comité de Ética Independiente ZUGUEME |  | Guatemala City  (Guatemala) |
|  | Hong Kong | 4761 | Joint Chinese University of Hong Kong- New territories East cluster Clinical Research Ethics Committee | NA | Hong Kong  (NA) |
|  | Hong Kong | 4762 | Kowloon West Cluster Research Ethics Committee | NA | Hong Kong  (NA) |
|  | Hungary | All sites/central Vote | Medical Research Council | Ethics Committee for Clinical Pharmacology | Budapest |
|  | Iceland | 4810 | The National Bioethics Committee |  | Reykajvik |
|  | India | 4823 | Institutional Ethical Review Board |  | Bangalore  (Karnataka) |
|  | India | 4827 | KRIMS Ethics Committee |  | Nagpur  (Maharashtra) |
|  | India | 4828 | Institutional Ethics Committee |  | Coimbatore  (Tamil Nadu) |
|  | India | 4830 | Institutional Ethics Committee |  | Visakhapatnam  (Andhra Pradesh) |
|  | India | 4831 | Fortis Hospital Ethics Committee |  | Kolkata  (West Bengal) |
|  | India | 4833 | Ethics Committee of Care Institute of Medical Sciences |  | Ahmedabad  (Gujarat) |
|  | India | 4835 | Drug Trial Ethics Committee |  | Ludhiana  (Punjab) |
|  | India | 4837 | Institutional Human Ethics Committee |  | Coimbatore  (Tamil Nadu) |
|  | India | 4838 | AMC MET Ethics Committee |  | Ahmedabad  (Gujarat) |
|  | India | 4840 | Paras Hospital Ethics Committee |  | Gurgaon  (Harayana) |
|  | India | 4841 | Institutional Ethics Committee |  | Hyderabad  (Andhra Pradesh) |
|  | India | 4843 | Getwell Institutional Ethics Committee (GIEC) |  | Nagpur  (Maharashtra) |
|  | Italy | 4890 | Comitato per la Sperimentazione Clinica dei Medicinali Area Vasta Nord | c/o Azienda Ospedaliero Universitaria Pisana di Pisa | Pisa |
|  | Italy | 4891 | Comitato Etico per le Sperimentazioni Cliniche della Provincia di Padova | C/O AOU Padova | Padova |
|  | Italy | 4892 | Comitato Etico Regionale Unico (CERU) | c/o Azienda Ospedalierouniversitaria | Udine |
|  | Italy | 4893 | Comitato Etico Area di Pavia | c/o Fondazione Irccs Policlinico San | Pavia |
|  | Italy | 4894 | Comitato Etico Seconda Universita' Degli Studi di Napoli | Aou Sunaorn | Napoli |
|  | Italy | 4895 | Comitato Etico Provinciale di Varese | c/o Ospedale di Circolo e | Varese |
|  | Italy | 4897 | Comitato Etico Dell'Azienda Ospedaliero-Universitaria Ospedali Riuniti |  | Foggia |
|  | Italy | 4898 | Comitato Etico Milano Area C | c/o Azienda Ospedaliera Ospedale Niguarda Ca' | Milano |
|  | Italy | 4899 | Comitato Etico per la Sperimentazione Clinica delle Province di Verona | C/O AOUI Verona | Verona |
|  | Italy | 4900 | Comitato Etico Provinciale di Reggio Emilia | c/o Azienda Ospedaliera Arcispedale S. Maria Nuova/IRCCS | Reggio emilia |
|  | Italy | 4901 | Comitato Etico Dell'IRCCS Fondazione Don Carlo Gnocchi di Milano | Centro S. Maria Nascente | Milano |
|  | Italy | 4902 | Comitato Etico Della Provincia di Modena | C/O Azienda Policlinico, | Modena |
|  | Italy | 4903 | Comitato Etico Aree Cremona, Mantova e Lodi | c/o AO di Cremona | Cremona |
|  | Italy | 4904 | Comitato Etico Regionale (CER) | c/o Azienda Ospedaliera Universitaria Ospedali Riuniti Umberto I-Lancisi-Gm Salesi di Ancona | Ancona |
|  | Italy | 4906;#4905 | Comitato Etico Centrale Dell'IRCCS Fondazione Salvatore Maugeri (IRCCS) di Pavia |  | Pavia |
|  | Italy | 4908 | Comitato Etico Dell`IRCCS Ospedale Oncologico di Bari |  | Bari |
|  | Italy | 4909 | Comitato Etico Regione Liguria | c/o IRCCS Aou San Martino - Ist Istituto Nazionale per la Ricerca Sul Cancro | Genova |
|  | Japan | 1009;#1011;#1014;#1020;#1006;#1012 | National Hospital Organization CRB | National Hospital Organization | Meguro-ku  (Tokyo) |
|  | Japan | 1000 | Nihon Koukan Hospital IRB | Nihon Koukan Hospital | Kawasaki  (Kanagawa) |
|  | Japan | 1001 | Tokyo-Eki Center-building Clinic IRB |  | Chuo-ku  (Tokyo) |
|  | Japan | 1002 | Nakatani Hospital IRB |  | Himeji  (Hyogo) |
|  | Japan | 1003 | JCHO Hokkaido Hospital IRB |  | Sapporo  (Hokkaido) |
|  | Japan | 1004 | Tenryu Hospital IRB |  | Hamamatsu  (Shizuoka) |
|  | Japan | 1005 | Kishiwada City Hospital IRB |  | Kishiwada  (Osaka) |
|  | Japan | 1007 | Sakaide City Hospital IRB |  | Sakaide  (Kagawa) |
|  | Japan | 1008 | Hamamatsu Rosai Hospital IRB |  | Hamamatsu  (Shizuoka) |
|  | Japan | 1010 | Matsusaka City Hospital IRB |  | Matsusaka  (Mie) |
|  | Japan | 1013 | Tosei General Hospital IRB |  | Seto  (Aichi) |
|  | Japan | 1015 | Obihiro Kokyukika Naika Hospital IRB |  | Obihiro  (Japan) |
|  | Japan | 1016 | Iwata City Hospital IRB |  | Iwata  (Shizuoka) |
|  | Japan | 1017 | Takamatsu Manicipal Hospital IRB |  | Takamatsu  (Kagawa) |
|  | Japan | 1018 | Daido Hospital IRB |  | Nagoya  (Aichi) |
|  | Japan | 1019 | Nagata Hospital IRB |  | Yanagawa  (Fukuoka) |
|  | Japan | 1021 | Harasanshin Hospital IRB |  | Fukuoka  (Fukuoka) |
|  | Japan | 1022 | Tohoku University Hospital IRB |  | Sendai  (Miyagi) |
|  | Japan | 1023 | Nihon University Hospitals' Joint IRB | Nihon University Hospitals | Itabashi-ku  (Tokyo) |
|  | Japan | 1024 | Juntendo University Hospitals IRB |  | Bunkyo-ku  (Tokyo) |
|  | Japan | 1025 | Hokkaido University Hospital IRB |  | Sapporo  (Tokyo) |
|  | Japan | 1026 | Fujita Health University hospital IRB |  | Toyoake  (Aichi) |
|  | Japan | 1027 | Kyoto University Hospital IRB |  | Kyoto  (Kyoto) |
|  | Japan | 1028 | ICR Clinical Research Tokyo Hospital IRB | ICR Clinical Research Tokyo Hospital | Shinjuku-ku  (Tokyo) |
|  | Japan | 1029 | Kimura Hospital IRB | Kimura Hospital | Ota-ku  (Tokyo) |
|  | Latvia | 1230;#1231;#1233 | P. Stradins Clinical University Hospital EC |  | Riga |
|  | Lithuania | 1250;#1251;#1252;#1253;#1254;#1255;#1257;#1259;#1260 | Lietuvos bioetikos komitetas (Lithuanian Bioethics Committee) |  | Vilnius |
|  | Mexico | 1280;#1284 | Comité Bioético para la Investigación Clínica S.C. | NA | Mexico City  (Mexico City) |
|  | Mexico | 1281 | Comité de Ética del Hospital General de México "Dr. Eduardo Liceaga" | Dirección de Investigación | Mexico City  (Mexico City) |
|  | Mexico | 1282 | Comité de Ciencia y Bioética en Investigación del Instituto Nacional de Enfermedades Respiratorias "Ismael Cosío Villegas" | NA | Mexico City  (Mexico City) |
|  | Mexico | 1283 | Comité de Ética del Hospital Ángel Leaño | NA | Guadalajara  (Jalisco) |
|  | Mexico | 1285 | Comité de Bioética en Investigación Christus Muguerza Hospital UPAEP | NA | Puebla  (Puebla) |
|  | Mexico | 1286 | Centro de Investigación Medico Biologica y Terapia Avanzada S.C. | NA | Guadalajara  (Jalisco |
|  | Netherlands | 1310;#1311;#1312;#1315;#1313;#1316;#1317;#1318;#1319;#All sites/central Vote | MEC-U (Medical Research Ethics Committees United) |  | Eindhoven |
|  | Norway | All sites/central Vote | Regionale komiteer for medisinsk og helsefaglig forskningsetikk (REK) | REK Sør-øst B | Oslo |
|  | Philippines | 1380 | Lung Center of the Philippines | Institutional Ethics Review Board | Quezon City  (Quezon Avenue Extension) |
|  | Philippines | 1381 | University of the Philippines Manila | Research Ethics Board | Manila  (Pedro Gil St. Ermita) |
|  | Philippines | 1382 | Mary Mediatrix Medical Center | Research Ethics Committee | Batangas, Lipa City  (J.P. Laurel Highway) |
|  | Poland | 1401;#1400;#1402;#1403;#1404;#1405;#1406 | Komisja Bioetyki Uniwersytetu Medycznego w Łodzi | NA | Łódź  (NA) |
|  | Portugal | 1430;#1431;#1432;#1433;#1434;#1435;#1436;#1437;#1438;#1439;#1440 | CEIC - Parque da Saúde de Lisboa | Not Applicable | Lisbon  (Lisbon) |
|  | Republic of korea | 1205 | Kyunghee University Medical Center | IRB | Seoul  (1, Heogi-dong, Dongdaemun-gu) |
|  | Republic of korea | 1207 | Seoul National University Hospital | IRB | Seoul  (103, Daehak-ro, Jongno-gu) |
|  | Republic of korea | 1200 | Korea University Anam Hospital | IRB | Seoul  (126-1, Anam-dong 5-ga, Sungbuk-gu) |
|  | Republic of korea | 1202 | The Catholic University of Korea Seoul St. Mary’s Hospital | IRB | Seoul  (505, Banpo-dong, Seocho-gu) |
|  | Republic of korea | 1203 | Chungang University Hospital | IRB | Seoul (224-1 Heukseok-dong, Dongjak-gu) |
|  | Republic of korea | 1204 | Soonchunhyang University Hospital_Bucheon | IRB | Gyeonggi-do  (1174, Jung-dong, Wonmi-gu, Bucheon-si) |
|  | Republic of korea | 1201 | The Catholic University of St Paul`s Hospital | IRB | Seoul  (Jeonnong 1-dong, Dongdaemun-gu) |
|  | Republic of korea | 1206 | The Catholic University of Korea Incheon St. Mary's Hospital | IRB | Incheon  (665-8, Bupyeong-dong, Bupyeong-gu) |
|  | Republic of korea | 1209 | Chonbuk National University Hospital | IRB | Chonbuk  (634–18, Geumam-dong, Deokjin-gu, Jeonju-si, Jeollabuk-do) |
|  | Romania | All sites/central Vote | Comisia Nationala de Bioetica a Medicamentului si Dispozitivelor Medicale |  | Bucharest |
|  | Russian federation | All sites/central Vote | Ethics committe of Russian Fediration |  | Moscow |
|  | Serbia | 1790 | EC of Clinical Centre Nis |  | Nis |
|  | Serbia | 1791 | EC of Clinical Centre Kragujevac |  | Kragujevac |
|  | Serbia | 1792 | EC of Clinical Centre of Serbia |  | Belgrade |
|  | Serbia | 1793 | EC of Clinical Hospital Centre Bezanijska Kosa |  | Belgrade |
|  | Slovakia | 1540;#1545;#1541;#1542;#1543;#1544;#1546;#1547;#1548;#1549;#1551;#1552;#1553;#1554;#1555;#1556 | Eticka komisia Kosickeho samospravneho kraja | Odbor Zdravotnictva | Kosice  (NA) |
|  | South africa | 1570;#1571;#1572;#1573;#1574;#1576;#1578;#1579;#1580 | Pharma Ethics Independant Research Ethics Committee |  | Pretoria  (Gauteng) |
|  | South africa | 1581 | University of Stellenbosch Human Research Ethics Committee | Division of research development and support | Cape Town  (Western Cape) |
|  | South africa | 1575 | University of Cppe Town Human Research Ethics Committee | Faculty of Health Sciences | Cape Town  (Western Cape) |
|  | Spain | 1602;#1601;#1603;#1604;#1605;#1607;#1610;#1611;#1612;#1614;#1613;#1617;#1616;#1618;#1620;#1623;#1624;#1626;#1631;#1630;#1629;#1628;#1627 | FUNDACIÓ UNIÓ CATALANA D’HOSPITALS | Comité Ético Investigación Clínica | Barcelona  (Barcelona) |
|  | Sweden | 1651;#1650;#1652;#1655;#1656;#1657 | Regionala etikprövningsnämnden i Lund | Box 133 | Lund |
|  | Taiwan | 1670 | Institutional Review Board | Taipei Veterans General Hospital | Taipei |
|  | Taiwan | 1671 | Research Ethics Committee | National Taiwan University Hospital | Taipei |
|  | Taiwan | 1672 | Research Ethics Review Committee | Far Eastern Memorial Hospital | Taipei |
|  | Taiwan | 1673 | Research Ethics Committee | China Medical University Hospital | Taichuang |
|  | Taiwan | 1674 | The Institution Review Board of Taichuang Veterans Gereral Hospital | Taichuang Veterans Gereral Hospital | Taichuang |
|  | Taiwan | 1675 | Institutional Review Board | Chang Gung Medical Foundation | Taoyuan County |
|  | Taiwan | 1676 | Institutional Review Board | National Cheng Kung University Hospital | Tainan |
|  | Taiwan | 1677 | Institutional Review Board | Chang Gung Medical Foundation | Taoyuan County |
|  | Thailand | 1751 | The Office of Human Research Ethics | Khon Kaen University | Khon Kaen |
|  | Thailand | 1752 | Research Ethics Committee | Faculty of Medicine,Chiang Mai University | Chiang Mai |
|  | Thailand | 1753 | The Research Ethics Committee | Faculty of Medicine, Prince of Songkla University | Songkla |
|  | Turkey | 1697;#1696;#1695;#1694;#1693;#1692;#1691;#1690 | Baskent University Medical Faculty Clinical Research Ethics Committee | - | Ankara  (Besevler) |
|  | United Kingdom | All sites/central Vote | Yorkshire & The Humber - Leeds West | TEDCO Business Centre | Rolling Mill Road  (Jarrow) |
|  | Argentina | 4003;#4008;#4011;#4014;#4017;#4019;#4023;#4027;#4029 | Comite Independiente de Etica para Ensayos en Farmacologia Clinica | NA | CABA  (Buenos Aires) |
